# Out-Patient Operations

**Published:** 1909-06-05

**Authors:** 


### RESIDENT MEDICAL OFFICERS' DEPARTMENT.

### OUT-PATIENT OPERATIONS.

THE out-patient operating theatre is nowadays an essential part of a modern general hospital, and it makes a large claim upon the time of the house surgeon. The variety and extent of the operations performed there would probably astonish one whose personal acquaintance with hospital routine closed, say, fifteen years ago. There are quite a number of operations for which the out-patient theatre or operating room is the proper place; but there are others of about the same extent and importance, for which it is not. In other words, the out-patient theatre has limitations, often transgressed by the inexperienced, which are worthy of definition and observance.

In the large general hospitals of big towns this department falls, as a rule, to the assistant house surgeon, for whom the assistant house physician acts as anæsthetist. Under this system both officials may gain experience of their respective duties, and acquire by humble beginnings dexterity and sound technique. In the main, also, it is for the best advantage of patients; for if senior house officers undertook these duties some more responsible tasks would fall to the lot of the juniors, where their inexperience would be a greater detriment.

Now aseptic surgery and the enormous improvements in surgical teaching which have taken place during the last few years have made it an every day occurrence for comparative novices to attempt fairly extensive operative surgery with highly successful results. And as far as the actual operation itself goes, the junior house surgeon at a hospital can usually be trusted to do excellent work in the out-patient theatre; since what he may lack in years he makes up for by energy and enthusiasm. But it sometimes happens that he undertakes tasks for which the out-patient department is not a fit place, and that his results suffer in consequence.

Let us first consider the preparation of the patient. At many hospitals he is given a printed notice filled in with the exact date and hour at which he is to attend, and enjoining on him the necessary purgation and abstinence from food for the required number of hours. Yet however explicit this leaflet may be there are many patients too careless to study it, too ignorant to understand it, or too incredulous to obey its directions. On no account should the operator or, better still, the anæsthetist fail to inquire the time of the last meal. To the working classes a meal means a somewhat extensive repast; a bun and a glass of milk for a child, or beer and bread and cheese for an adult, are regarded as too insignificant to deserve mention. Another point of importance is to ensure that the patient has an opportunity for micturition immediately before entering the operating room.

Anæsthetic after-effects must be obviated if possible, since the patient has to return home on the day of operation. By preference gas should always be used, unless a local anæsthetic will suffice; failing this, ethyl chloride may be enough for many small operations which yet cannot be completed satisfactorily under gas. There are, however, some very brief operations for which gas is unsuitable. Thus any condition about the anus, such as a thrombosed pile needing incision, usually calls for etherisation, no matter how brief the operation; for under gas the first manipulation of this region is apt to produce almost opisthotonic spasms. For breaking down adhesions, also, gas is seldom satisfactory.

For longer operations the gas-ether sequence is advisable. The effects of a short etherisation pass off fairly quickly, especially in a robust man accustomed to alcohol; and there are other reasons for the choice of ether in preference to chloroform or a chloroform mixture. Ether by the open method is hardly likely to be necessitated in the out-patient theatre by the patient's condition, but there would be no other objection to it than the loss of valuable time which it entails during induction. In certain circumstances gas and ether are both contra-indicated; the general principles of selection of an anæsthetic must be remembered, but amongst out-patients chloroform is most frequently indicated for suppuration in the neck and about the floor of the mouth.

Turning now to the operations themselves, it is of very doubtful expediency to do any but the simplest upon the lower limbs. An ingrowing toe-nail or a perforating ulcer may legitimately be attacked; and, of course, abscesses if not too large. But cellulitis, tenotomy, and patellar bursæ call for in-patient treatment. However carefully a leg may be splinted, it cannot receive proper rest, when on the day of operation the patient has to get home, where he cannot be nursed.

Once the broad principles of selection are grasped, the house-surgeon whose enterprise and resource are equalled by his care in preparation and after-treatment, may add appreciably to his list of out-patient operations. Thus excision of the interphalangeal joint for hammer toe is quite feasible if a neat metal splint to go under the toe is ready, and a generous dressing is encased in a large carpet slipper. Foreign bodies—chiefly pins and needles trodden on—are also practicable if located immediately beforehand by the *x*-rays.

Of buboes and phimosis it is hardly necessary to speak. They constitute a large percentage of out-patient work; and the latter as a whole are as satisfactory as the former are disappointing. Mammary abscess should not, as a rule, be opened under gas or ethyl chloride. There are few suppurative lesions so apt to become intractable if not thoroughly dealt with at the first operation. Full etherisation and methodical incision of every pocket of the abscess which can be found, with free drainage and constant care in the after-treatment will generally be rewarded with success. Tonsils and adenoids form another of the principal groups of out-patient cases. To attempt both under gas only is not very much to be recommended, even for the practised operator; but ethyl chloride generally gives a long enough anæsthesia for the purpose. Iced water, with which to sponge the child's face after the operation, is useful in hæmorrhage.

On the arms the house surgeon may perform a number of operations which are contra-indicated on the leg; for here complete rest ought to be ensured by appropriate splinting, and the inclusion of abundant wool in the dressings. Thus an olecranon bursa may well be excised in the out-patient theatre, though the corresponding condition of the patella ought to be admitted to a bed. So, too, needles may be sought for even when quite deep in the hand or arm, whereas only those which are fairly superficial should be attempted in the lower limb. Ganglion is another condition which may be radically treated if the operator has complete confidence in his aseptic or antiseptic technique; and amputations of the fingers, suture of tendons after injury, compound fractures of the bones of the hand, and so on, may be fearlessly operated on. So may, as a rule, the small fibro-adenomata of the breast which occur in women about the ages of twenty to twenty-three; but these tumours should always be submitted to the pathologist for section. Lipomata, even of large size, are also generally suitable for excision, and should either be drained for forty-eight hours or the recesses of the cavity drawn together with a buried suture. There are very many other operations of minor surgery for which the out-patient theatre is nowadays appropriate, but even the brief list enumerated includes many which only a few years ago no one would have dreamt of undertaking, except upon in-patients.

